# Modeling the Effect of Material Viscoelasticity on the Dielectric Permittivity of Deformed Elastomers

**DOI:** 10.3390/polym16010113

**Published:** 2023-12-29

**Authors:** Xianghe Zheng, Jianyou Zhou

**Affiliations:** School of Science, Harbin Institute of Technology, Shenzhen 518055, China

**Keywords:** dielectric elastomers, dielectric permittivity, material viscoelasticity, polarization, rate-dependency

## Abstract

Elastomers, as a typical category of soft dielectrics, have shown great potential for developing stretchable electronics and soft transducers. However, the performance of dielectric elastomers (DEs) is susceptible to the dielectric permittivity of the material, whether as insulators or actuators. On the other hand, experiments suggest that the material viscoelasticity significantly influences the dielectric permittivity of DEs. Based on the theory of finite-deformation viscoelasticity, this work adopts the Brillouin function to develop a modeling framework to examine the effect of material viscoelasticity on the dielectric permittivity for the first time. A comparison of the data fitting results between the models with and without consideration of the material viscoelasticity is presented. Simulation results also reveal that the viscous network of the elastomer exerts a mitigation effect on the decrease in the dielectric permittivity when the material is deformed. Furthermore, it is found that the loading rate is a key parameter that strongly affects the dielectric permittivity, mainly through the inelastic deformation.

## 1. Introduction

In recent years, with the development of stretchable electronics and soft robotics, soft dielectrics, such as dielectric elastomers (DEs), have attracted much interest in the research community. DEs have been developed as actuators [1,2], tunable optic devices [3,4], energy harvesters [5,6], etc. Whether for insulators or transducers, the dielectric permittivity of dielectric elastomers is the key factor that significantly influences their overall performance. For example, in the signal transmission process, the delay of signal strongly depends on the dielectric permittivity of the material [7,8]. Also, as the Maxwell pressure of a dielectric elastomer membrane is proportional to the dielectric permittivity, the actuation performance of DE actuators is strongly affected by the dielectric permittivity [9,10,11].

Although dielectric permittivity is also known as a dielectric constant sometimes, experiments have suggested that the dielectric permittivity of elastomers is not constant at all in most cases. In fact, the dielectric permittivity of elastomers is susceptible to a variety of factors, such as frequency of the applied voltage, temperature, relative humidity, and, especially, deformation [12]. Particularly, the deformation-dependent dielectric permittivity of DEs makes controlling their actuation rather challenging. To tackle this issue, much attention has been given to the deformation-dependent dielectric permittivity of widely used dielectric elastomers, such as the very high bond (VHB) elastomers by 3M corporation (St. Paul, MN, USA) [13,14,15]. An early experimental study by Kofod et al. showed that the dielectric permittivity of VHB elastomers almost linearly dropped with the pre-stretch ratio to a certain level (about 5% at the equi-biaxial stretch ratio of 5) [16]. On the other hand, Choi et al. reported that the dielectric permittivity of VHB elastomers dramatically decreased as the equi-biaxial stretch ratio increased (over 50% at a stretch ratio of 4) [17]. Wissler and Mazza also reported a linear reduction in the dielectric permittivity with the stretch ratio and to a large extent (around 50% at a stretch ratio of 5) [18]. Tröls et al. presented a comparison of the deformation-dependent dielectric permittivity between VHB elastomers and natural rubbers (ZruElast 1040), showing that the effect of the deformation on the dielectric permittivity of natural rubbers was less pronounced [19]. Although the experimental data reported by different studies is rather scattered, a consensus has been reached that the dielectric permittivity of the elastomers decreases with the increasing in-plane stretches. The scatter of the data is mostly attributed to the difference in experiment setup, environmental conditions, and materials of the electrodes. In fact, the material viscoelasticity is also an important factor that is usually overlooked (elastomers are more or less viscous) [20,21,22]. Hence, to eliminate the effect of material viscoelasticity, Li et al. later conducted measurements of the dielectric permittivity seven days after the fabrication of samples [23].

Other than experimental studies, theoretical models have also been proposed to capture the deformation-dependence of the dielectric permittivity. In the early work of Zhao and Suo, a phenomenological model was used to describe the quasilinear dielectric behavior of elastomer membranes subjected to large deformation [24]. Later, adopting the Brillouin function, Li et al. proposed a model, particularly for the conditional (under mechanical constraints) polarization of dielectric elastomers [23]. Based on statistical mechanics, Jiménez and McMeeking established a correlation between the dielectric permittivity of an elastomer and the general state of deformation [25]. Schlögl and Leyendecker proposed a polarization-based lumped parameter model for the electromechanical coupled dielectric permittivity [26]. Nevertheless, most existing studies that consider the deformation-dependence of the dielectric permittivity of elastomers assume purely elastic deformation of the material. In other words, the material viscoelasticity is ignored. This assumption may hold when the deformation is small or quasi-static.

However, the advantage of dielectric elastomer actuators mainly lies in their capability to produce large deformations. Also, as actuators, DEs are usually subjected to dynamic loads. Therefore, examining the effect of the material viscoelasticity on the dielectric permittivity is of importance. From a physical point of view, the dielectric permittivity is a parameter that characterizes the degree of polarization of the material. It is well-established that polarization is deformation-dependent [27]. Meanwhile, for elastomers, their deformation is more or less viscoelastic. Hence, in the present contribution, we first explore the effect of the material viscoelasticity on the polarization of the dipoles within the elastomer and then reveal the corresponding influence on the dielectric permittivity.

## 2. Theory and Methods

**Figure 1:**
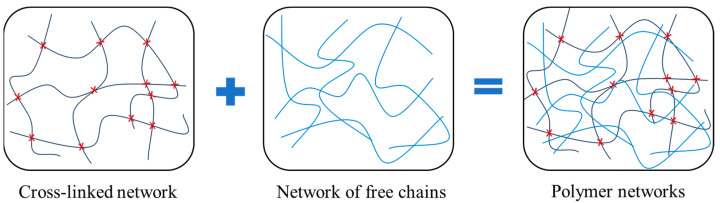
Polymer networks consist of cross-linked (red crosses) polymer chains and free chains.

**Figure 2:**
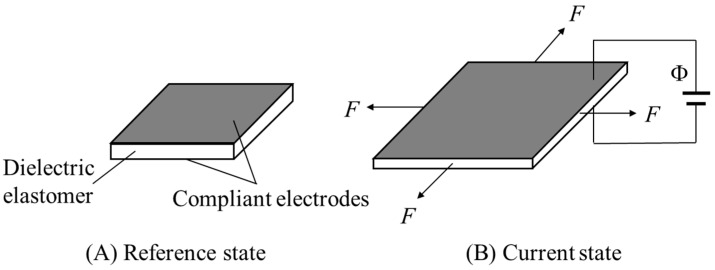
A dielectric elastomer membrane coated with compliant electrodes on both sides. (**A**) The reference state and (**B**) the current state when the elastomer is subjected to equi-biaxial forces *F* and voltage Φ.

Since dielectric permittivity is essentially a parameter that characterizes the polarization of the material, we first consider the polarization of an elastomer membrane in this section. Elastomers are generally formed by polymer networks of highly mobile and flexible polymer chains [20,28]. As shown in Figure 1, we simply consider that the polymer networks of elastomers can be separated into a cross-linked network and a viscous network. On the one hand, the hyperelasticity (capability of sustaining large deformation) of elastomers is mainly attributed to the cross-linking of the polymer chains (cross-linked network). On the other hand, owing to the diffusion of the free polymer chains (viscous network), elastomers are more or less viscous, exhibiting a time-dependent or rate-dependent response. Considering an elastomer membrane shown in Figure 2, the average dipole moment $\langle \mu_{e}\rangle$ in the thickness direction for a unit volume can be expressed as

(1) $$\langle \mu_{e}\rangle =\chi \langle \mu_{c}\rangle +\left(1-\chi \right)\langle \mu_{v}\rangle ,$$

where parameter $\chi =n_{c}/\left(n_{c}+n_{v}\right)$ is the volume fraction of the cross-linked network, *n*_c_ is the number of cross-linked chains per unit volume, *n***_v_** is the number of free chains per unit volume, and $\langle \mu_{c}\rangle$ and $\langle \mu_{v}\rangle$ are the average dipole moments of a cross-linked chain and a free chain, respectively. It should be noted that the shear moduli of the cross-linked network and the viscous network are $G_{c}=n_{c}k_{b}T$ and $G_{v}=n_{v}k_{b}T$, where *k*_b_ is the Boltzmann constant, and *T* is the temperature. “c” and “v” represent “cross-linked” and “viscous”, respectively [28].

The Brillouin function $B_{J}\left(x\right)$ is a commonly used mathematical model to describe the magnetization of paramagnetic and ferromagnetic materials [29,30]. Here, considering the similarity between magnetic and electric polarization (dipoles), we adopt the Brillouin function to describe the polarization of the electric dipoles in a uniform electric field *E*, i.e.,

(2) $$\langle \mu \rangle =\mu B_{J}\left(\frac{\mu E}{k_{b}T}\right)=\mu \left[\frac{2J+1}{2J}\coth\left(\frac{2J+1}{2J}\frac{\mu E}{k_{b}T}\right)-\frac{1}{2J}\coth\left(\frac{1}{2J}\frac{\mu E}{k_{b}T}\right)\right],$$

where $\mu E/k_{b}T$ denotes the internal energy. Here, *J* is a negative material parameter indicating the number of states that the electric dipoles may be in, before the electric field is applied [23]. In other words, *J* is an indicator of the level of the mechanical constraint on the polarization of the electric dipoles. When *J* approaches negative infinity, the Langevin function $L\left(x\right)=\coth\left(x\right)-1/x$ is recovered, in which case the elastomer becomes an ideal dielectric, and the dipoles respond freely to the external electric field (ideal dielectrics, without any constraints on their polarization) [24].

Generally, for polymeric dielectrics, $\mu E/k_{b}T\ll 1$ [23] and the Brillouin function is reduced to

(3) $$B_{J}\left(\frac{\mu E}{k_{b}T}\right)=\frac{1}{3}\left(\frac{J+1}{J}\right)\frac{\mu E}{k_{b}T}.$$

With Equations (2) and (3), Equation (1) can be re-written as

(4) $$\langle \mu_{e}\rangle =\chi \left(\frac{J_{c}+1}{J_{c}}\right)\frac{\mu_{c}^{2}E}{3k_{b}T}+\left(1-\chi \right)\left(\frac{J_{v}+1}{J_{v}}\right)\frac{\mu_{v}^{2}E}{3k_{b}T},$$

where *J*_c_ and *J*_v_ represent the level of constraint on the cross-linked network and the viscous network, respectively.

Then, the electric displacement *D* in the thickness direction is expressed as

(5) $$D=\varepsilon_{0}E+N\langle \mu_{e}\rangle =\varepsilon_{0}E+N\chi \left(\frac{J_{c}+1}{J_{c}}\right)\frac{\mu_{c}^{2}E}{3k_{b}T}+N\left(1-\chi \right)\left(\frac{J_{v}+1}{J_{v}}\right)\frac{\mu_{v}^{2}E}{3k_{b}T},$$

where $N\langle \mu_{e}\rangle =P$ is the polarization in the thickness direction, *N* is the polymerization degree of chains, and *ε*_0_ is the vacuum permittivity. As the dielectric permittivity *ε* of the material is defined by $D=\varepsilon \varepsilon_{0}E$,

(6) $$\varepsilon =\frac{D}{\varepsilon_{0}E}=1+\chi \left(\frac{J_{c}+1}{J_{c}}\right)\frac{\mu_{c}^{2}}{3\varepsilon_{0}k_{b}T/N}+\left(1-\chi \right)\left(\frac{J_{v}+1}{J_{v}}\right)\frac{\mu_{v}^{2}}{3\varepsilon_{0}k_{b}T/N}.$$

When the elastomer membrane undergoes deformation, especially large deformation, the rotation of the dipoles in the electric field is constrained by the deformation to some extent. Hence, the indicators of constraints, *J*_c_ and *J*_v_, should be functions of the deformation.

Take the elastomer membrane in Figure 2 for example, the elastomer deforms from the reference state (Figure 2A) to the current state (Figure 2B) when subjected to equi-biaxial forces *F* and a voltage Φ between the compliant electrodes. An electric field *E* is induced by the applied voltage in the thickness direction. The total deformation gradient of the current state with respect to the reference state is given as

(7) $$F=\left[\begin{array}{ccc} \lambda & 0 & 0\\ 0 & \lambda & 0\\ 0 & 0 & \lambda^{-2} \end{array}\right],$$

where *λ* is the equi-biaxial stretch ratio. Also, as commonly treated in the literature, rubber-like materials, such as elastomers, are usually assumed to be incompressible [31,32], which gives $\det\left(F\right)=1$. For viscoelastic elastomers, the total deformation gradient can be multiplicatively split into two parts, i.e., $F=F^{e}F^{i}$, where the elastic and inelastic deformation gradients are given as

(8) $$F^{e}=\left[\begin{array}{ccc} \lambda^{e} & 0 & 0\\ 0 & \lambda^{e} & 0\\ 0 & 0 & {\left(\lambda^{e}\right)}^{-2} \end{array}\right]\;\mathrm{and}\;F^{i}=\left[\begin{array}{ccc} \lambda^{i} & 0 & 0\\ 0 & \lambda^{i} & 0\\ 0 & 0 & {\left(\lambda^{i}\right)}^{-2} \end{array}\right],$$

where $\lambda^{e}$ and $\lambda^{i}$ are the elastic and inelastic stretch ratios, respectively, noting that $\det\left(F^{e}\right)=\det\left(F^{i}\right)=1$ for incompressible materials [21,33].

Correspondingly, the strain energy density *W*_s_ of the elastomer can be split into two parts, i.e., strain energy densities *W*_c_ and *W*_v_ of the cross-linked network and the viscous network, respectively [21,33]. Moreover, elastic and inelastic deformations are linked by the thermodynamics evolution equation, namely,

(9) $$-\frac{1}{2}F\frac{d\left[{\left(C^{i}\right)}^{-1}\right]}{dt}F^{T}\cdot {\left(b^{e}\right)}^{-1}=\gamma^{-1}:\sigma^{\mathrm{NEQ}},$$

where $b_{}^{e}=F_{}^{e}{\left(F_{}^{e}\right)}^{T}$, $C_{}^{e}={\left(F_{}^{e}\right)}^{T}F_{}^{e}$, $C_{}^{i}={\left(F_{}^{i}\right)}^{T}F_{}^{i}$, and the stress of the viscous network $\sigma^{{}_{\mathrm{NEQ}}}=2F_{}^{e}\frac{\partial W_{v}\left(F^{e}\right)}{\partial C_{}^{e}}{\left(F_{}^{e}\right)}^{T}$. Also, $\gamma^{-1}=\frac{1}{2\eta }\left(I^{4}-\frac{1}{3}I⊗I\right)$ is an isotropic rank-four mobility tensor, and *η* is the viscosity of the elastomer [33]. Here, “NEQ” represents “non-equilibrium”, as the viscous network is usually in a non-equilibrium state. Adopting the Gent hyperelastic model [34] as the strain energy density functions of both the cross-linked and viscous networks, we have

(10) $$W_{c}=-\frac{G_{c}J_{\lim}^{}}{2}\ln\left(1-\frac{\lambda_{1}^{2}+\lambda_{2}^{2}+\lambda_{3}^{2}-3}{J_{\lim}^{}}\right),$$

(11) $$W_{v}=-\frac{G_{v}J_{\lim}^{}}{2}\ln\left[1-\frac{{\left(\lambda_{1}^{e}\right)}^{2}+{\left(\lambda_{2}^{e}\right)}^{2}+{\left(\lambda_{3}^{e}\right)}^{2}-3}{J_{\lim}^{}}\right],$$

where $\lambda_{i}\;\left(i=1,\;2,\;3\right)$ and $\lambda_{i}^{e}\;\left(i=1,\;2,\;3\right)$ are the principal stretches of the cross-linked and viscous network, respectively. Here, $J_{\lim}^{}=3(N-1)$ are material constants associated with the extensibility of the elastomer [35]. For equi-biaxial deformation, *λ*_1_ = *λ*_2_ = *λ* and *λ*_3_ = *λ*^−2^. Therefore, combining Equations (7)–(9) and (11), we obtain

(12) $$\frac{d\lambda^{i}}{dt}=\frac{J_{\lim}^{}\lambda^{i}\left[\lambda^{2}{\left(\lambda^{i}\right)}^{-2}-\lambda^{-4}{\left(\lambda^{i}\right)}^{4}\right]}{6\tau \left[J_{\lim}^{}-2\lambda^{2}{\left(\lambda^{i}\right)}^{-2}-\lambda^{-4}{\left(\lambda^{i}\right)}^{4}+3\right]},$$

where $\tau =\eta /G_{v}$ is defined as the relaxation time.

Now that *J*_c_ and *J*_v_ are functions associated with the deformation, we prescribe

(13) $$J_{c}=\alpha \ln\left(\frac{2\lambda^{2}+\lambda^{-4}-3+\delta }{J_{\lim}^{}}\right)-1,$$

(14) $$J_{v}=\alpha \ln\left[\frac{2{\left(\lambda^{e}\right)}^{2}+{\left(\lambda^{e}\right)}^{-4}-3+\delta }{J_{\lim}^{}}\right]-1=\alpha \ln\left[\frac{2\lambda^{2}{\left(\lambda^{i}\right)}^{-2}+\lambda^{-4}{\left(\lambda^{i}\right)}^{4}-3+\delta }{J_{\lim}^{}}\right]-1,$$

where *α* is a scaling parameter that controls the variation of *J*_c_ and *J*_v_ when the material is under constraint (deformed), which needs to be determined by data fitting [23]. Here, *δ* is a sufficiently small number to ensure that the variable of the logarithmic function is positive. From Equations (6), (13) and (14), it can be seen that the dielectric permittivity is governed by both the elastic and inelastic deformation. Moreover, from Equation (12), the inelastic deformation is time-dependent and rate-dependent, thus resulting in time-dependent and rate-dependent dielectric permittivity.

## 3. Results and Discussions

As VHB elastomers (by 3M corporation, St. Paul, MN) are widely used dielectric elastomers in soft transducers, we will take VHB 4910 elastomers (1 mm in thickness) as a case study in this section. The dielectric permittivity of VHB 4910 has been measured in a number of studies in the literature. Table 1 shows the dielectric permittivity of VHB 4910 elastomers subjected to equi-biaxial load from four existing studies. More data regarding the dielectric permittivity of VHB elastomers (including VHB 4905) can be found in Ref. [12]. Table 1 shows that the data are rather scattered, which may be linked to multiple factors, such as the experiment setup, the electrode material, the test frequency, etc. However, attention was not particularly given to the effect of material viscoelasticity in most studies. In fact, measurements conducted at different times and moments after the fabrication of a sample usually result in different dielectric permittivity values. Therefore, ignoring the effect of the material viscoelasticity may also be a main reason for the scatter of the obtained data. To avoid the viscoelastic effect on the dielectric permittivity, Li et al. tried conducting measurements seven days after the fabrication of samples [23].

When the material viscoelasticity is eliminated in the modeling, *χ* = 1, and the second term in both Equations (4) and (6) is eliminated, which is equivalent to a purely elastic case, i.e.,

(15) $$\varepsilon =1+\left[\left(J_{c}+1\right)/J_{c}\right]{\bar{\mu }}_{c}^{2}/3.$$

**Figure 3:**
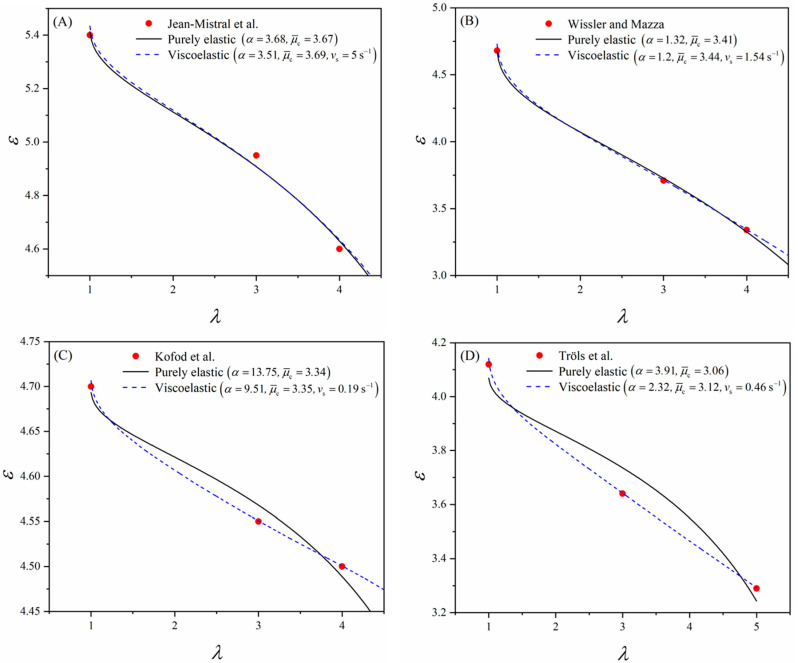
The dielectric permittivity is a function of the stretch ratio. Data from the works of (**A**) Jean-Mistral et al. [12], (**B**) Wissler and Mazza [18], (**C**) Kofod et al. [16], and (**D**) Tröls et al. [19]. The data are fitted using Equations (15) and (16), representing the purely elastic (black solid curve) and viscoelastic case (blue dash curve), respectively.

Here, owing to the lack of information on the chemical composition of VHB elastomers, the dipole moment is normalized as ${\bar{\mu }}_{c}=\mu_{c}/\sqrt{\varepsilon_{0}k_{b}T/N}$ and used as a parameter for data fitting. Then, the dielectric permittivity of the material is simply governed by the elastic deformation. Moreover, to avoid voltage-induced deformation, it is assumed that the testing voltage is sufficiently low (e.g., Φ = 1 V is commonly used in most studies). Figure 3 shows the fitting of Equation (15) against the data in Table 1 when *χ* = 1. The fitted parameters *α* and ${\bar{\mu }}_{c}$ are also given in Figure 3 (purely elastic case). Without considering the material viscoelasticity, Equation (15) can still describe the overall trend of the variation of the dielectric permittivity (i.e., the dielectric permittivity decreases with the equi-biaxial stretch ratio), while pronounced differences between some fitted curves and the experimental data can be observed (e.g., Figure 3C,D).

In order to take the material viscoelasticity into account, the calibrated material parameters for VHB 4910 elastomers are adopted in the viscoelastic modeling framework, e.g., $J_{\lim}^{}=90$, $G_{c}=25\;\mathrm{kPa}$, $G_{v}=70\;\mathrm{kPa}$, $\chi =G_{c}/\left(G_{c}+G_{v}\right)=0.263$, $\varepsilon_{0}=8.85\times 10^{-12}\;F/m$, and τ=50 s [36]. This is because the time moments for the measurements were not reported in the abovementioned studies (Table 1). Here, we consider a scenario in which the samples are stretched at a constant stretching rate to reach the prescribed stretch ratios (Table 1) to account for the time-dependent effect. Then, the stretching rate $v_{s}=d\lambda /dt$ (essentially strain rate) is used as a fitting parameter. In addition, it is assumed that the chemical compositions of the cross-linked chains and free chains are similar, which leads to ${\bar{\mu }}_{c}={\bar{\mu }}_{f}$. Then, Equation (6) is reduced to

(16) $$\varepsilon =\frac{D}{\varepsilon_{0}E}=1+\left[\chi \left(\frac{J_{c}+1}{J_{c}}\right)+\left(1-\chi \right)\left(\frac{J_{v}+1}{J_{v}}\right)\right]\frac{{\bar{\mu }}_{c}^{2}}{3}.$$

Combining Equations (12)–(14) and (16), the fitting of Equation (16) against the experimental data is also depicted in Figure 3 (viscoelastic case). The experimental data are well captured by Equation (16). However, due to the lack of data considering dynamic deformation, particular experiments could be designed to further examine the proposed modeling framework in future studies.

**Figure 4:**
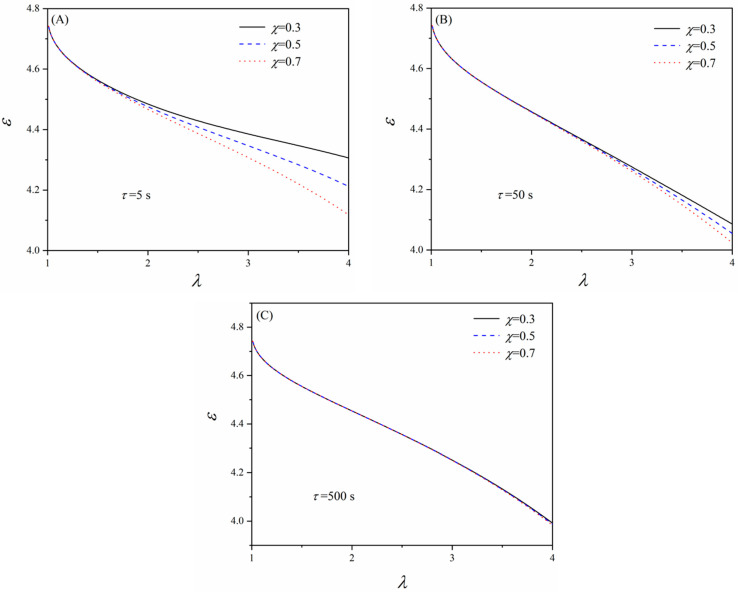
Variation of the dielectric permittivity with the equi-biaxial stretch ratio *λ* for different *χ* and *τ*. (**A**) *τ* = 5, (**B**) *τ* = 50, and (**C**) *τ* = 500.

To further explore the effect of the material viscoelasticity on the dielectric permittivity of elastomers, Figure 4 depicts the variation of the dielectric permittivity as a function of the fraction *χ* and relaxation time *τ*, which are the typical material parameters that reflect the viscoelasticity. Existing studies have suggested that elastomers may process multiple relaxation times depending on the loading conditions [36,37]. Correspondingly, multiple viscous networks should be considered to consider the multiple relaxation times. However, for simplicity, a single relaxation time is assumed in this work. Adopting multiple viscous networks may increase the model’s capability of capturing experimental data for more complex loading conditions. Other parameters are set as $v_{s}=0.5\;s^{-1}$, *α* = 3, and ${\bar{\mu }}_{c}={\bar{\mu }}_{f}=3.4$. For a certain relaxation time and a stretch ratio, e.g., *τ* = 5 s and *λ* = 3 (Figure 4A), the dielectric permittivity increases as the volume fraction *χ* decreases. Since *χ* represents the volume fraction of the cross-linked network, the effect of the viscous network on the dielectric permittivity becomes more dominant when *χ* decreases. Also, the mechanical constraints on the viscous network relax over time owing to the diffusion of the free chains. The dipoles in the viscous network will eventually be free to rotate under polarization if sufficient time is given. Therefore, the viscous network mitigates the decrease in the dielectric permittivity as the material deforms. However, comparing Figure 4A–C, it can be seen that the effect of this mitigation weakens when the relaxation time of the material rises. In Figure 4C, the three curves almost collapse onto one curve since the viscous network relaxes too slowly (a larger *τ* indicates a slower relaxation).

**Figure 5:**
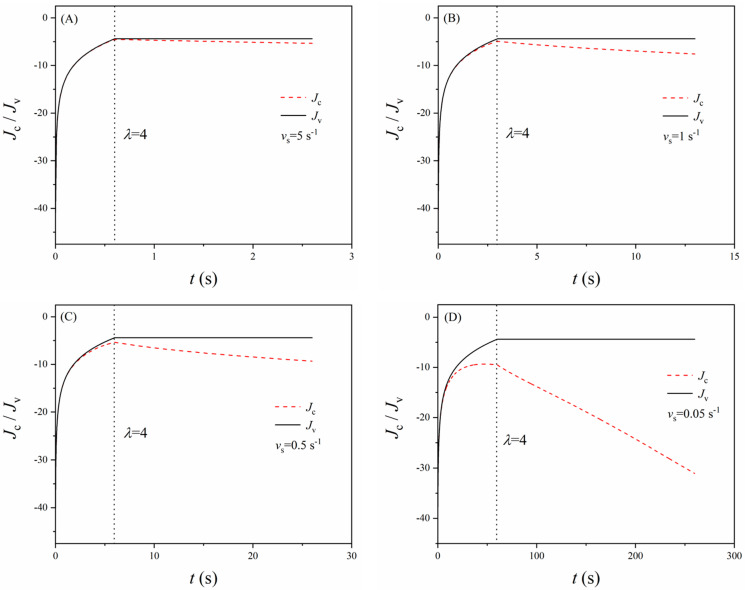
Change of parameters *J*_c_ and *J*_v_ with time as the loading rate (stretching rate) varies. (**A**) *v*_s_ = 5 s^−1^, (**B**) *v*_s_ = 1 s^−1^, (**C**) *v*_s_ = 0.5 s^−1^, and (**D**) *v*_s_ = 0.05 s^−1^.

**Figure 6:**
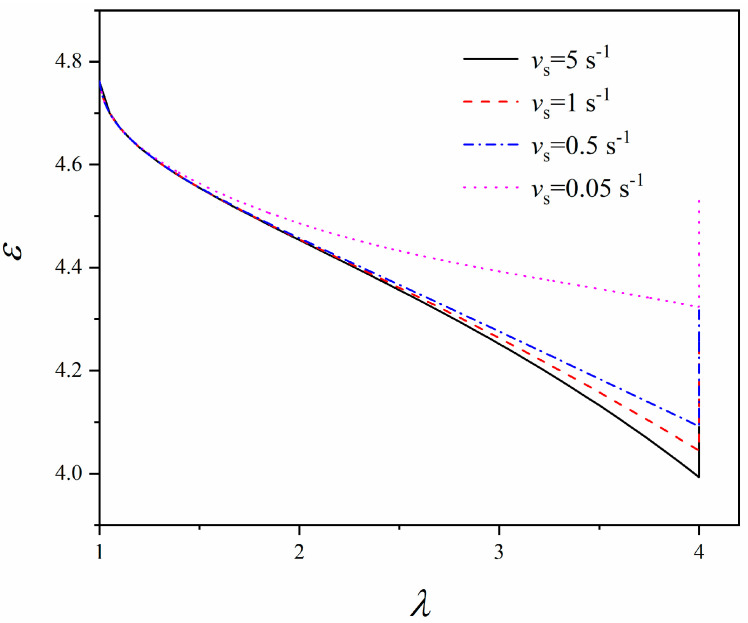
Dielectric permittivity versus the equi-biaxial stretch ratio at different loading rates (i.e., *v*_s_ = 5 s^−1^, 1 s^−1^, 0.5 s^−1^, and 0.05 s^−1^).

Another factor that strongly influences the relaxation of the viscous networks is the loading rate (stretching rate) since the loading rate directly affects the inelastic stretch ratio *λ*^i^ and thus *J*_v_ (Equations (12) and (14)). In addition, from Equation (13), it can be noted that *J*_c_ is independent of the loading rate. To examine the effect of the loading rate, we revisit a common situation in most experiments. The elastomer membrane is stretched from *λ* = 1 to *λ* = 4 at different loading rates, then the deformation is maintained thereafter. Similarly, *α* = 3, ${\bar{\mu }}_{c}={\bar{\mu }}_{f}=3.4$ and the material parameters of VHB 4910 elastomers are adopted here. As for the stretching rates, the typical values are chosen for a case study (i.e., *v*_s_ = 5 s^−1^, 1 s^−1^, 0.5 s^−1^, and 0.05 s^−1^). Figure 5 illustrates the change in *J*_c_ and *J*_v_ with time as the loading rate varies. Before reaching *λ* = 4, the change in *J*_v_ follows the trend of *J*_c_, except when the loading rate is rather slow (e.g., *v*_s_ = 0.05 s^−1^). For *v*_s_ = 0.05 s^−1^, *J*_v_ actually slightly drops after reaching a peak because the material relaxes faster at large deformation, and there is sufficient time for relaxation when the loading rate is slow. This is also the reason for the larger difference between *J*_c_ and *J*_v_ at slower loading rates. After reaching *λ* = 4, *J*_c_ remains the same since the deformation is maintained, while *J*_v_ linearly decreases with time as the relaxation of the material continues. Since *J*_c_ and *J*_v_, in essence, are indicators of the mechanical constraints on the polarization of the dipoles, the rise of *J*_c_ and *J*_v_ reflects stronger mechanical constraints on the polarization and vice versa. Owing to the diffusion of the viscous network, the material relaxes, and the mechanical constraints on the polarization of the viscous network weaken with time, which leads to a decrease in *J*_v_. Figure 6 shows the variation of the dielectric permittivity as a function of the stretch ratio at different loading rates. A lower loading rate results in a higher dielectric permittivity at a certain stretch ratio. Moreover, the range of the variation becomes narrower as the loading rate decreases, which is desirable for DE actuators from a control perspective. When the deformation is maintained at a stretch ratio of 4, the dielectric permittivity rises with time and will eventually approach to a steady state corresponding to $\varepsilon =1+\left[\chi \left(1-1/J_{c}\right)+1-\chi \right]{\bar{\mu }}_{c}^{2}/3$.

**Figure 7:**
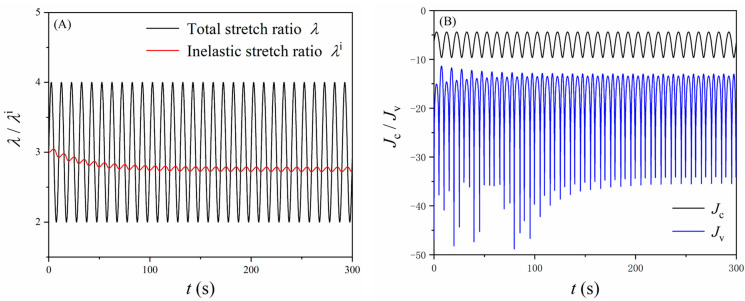
The dielectric elastomer membrane under equi-biaxial cyclic mechanical load (*f* = 0.1 Hz). (**A**) variation of *λ* and *λ*^i^ and (**B**) variation of *J*_c_ and *J*_v_.

**Figure 8:**
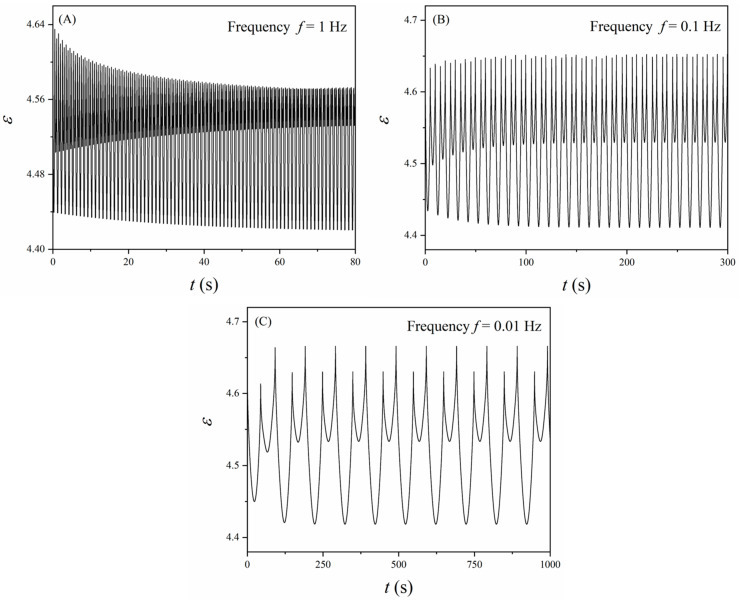
Variation of the dielectric permittivity when the DE membrane is subjected to the cyclic mechanical load of different loading frequencies. (**A**) *f* = 1 Hz, (**B**) *f* = 0.1 Hz, and (**C**) *f* = 0.01 Hz.

In real-world applications, dielectric elastomers are usually pre-stretched to avoid loss-of-tension and subjected to cyclic loads (e.g., DE oscillators and resonators [10]). Here, for example, we examine a common case that the DE membrane is equi-biaxially pre-stretched to *λ* = 3 and then subjected to cyclic mechanical load, i.e., $\lambda =A\sin\left(2\pi ft\right)+B$, where the amplitude *A* = 1, the bias *B* = 3, and the frequency *f* = 0.1 Hz. Also, it is considered that the DE membrane is fully relaxed (*λ* = *λ*^i^ = 3 at *t* = 0) before the cyclic mechanical load is applied. Figure 7A depicts the change of the stretch ratios *λ* and *λ*^i^ with time. The change of *λ*^i^ follows the trend of *λ*, while its amplitude is much smaller owing to the relatively high loading frequency. Also, as the loading cycles continue, the cycles of the inelastic stretch ratio *λ*^i^ become steady. Figure 7B illustrates the variation of parameters *J*_c_ and *J*_v_ with time. In this case, unlike the results shown in Figure 5, the starting points of *J*_c_ and *J*_v_ differ. Moreover, the value of *J*_c_ is always higher than that of *J*_v_, which indicates that *J*_c_ is the dominant constraint (Equation (16)) in such cases. Similar to the response of *λ*^i^, the cycles of *J*_v_ become identical after a few cycles as the cycles of *λ*^i^ become steady. Of course, it is expected that the responses of both *λ*^i^ and *J*_v_ also depend on the frequency of the mechanical load since they are affected by the material viscoelasticity. The responses of both *λ*^i^ and *J*_v_ are eventually reflected in the variation of the dielectric permittivity *ε*. Figure 8 shows the variation of *ε* under cyclic mechanical loading conditions, where the typical frequencies are selected as *f* = 1 Hz, 0.1 Hz, and 0.01 Hz, respectively. First, as the loading frequency increases, it takes longer for the dielectric permittivity to reach a steady cycle. Moreover, a different loading frequency leads to a different pattern of the response of the dielectric permittivity, which could be further utilized to tackle different tasks in particular applications.

## 4. Conclusions

Based on the theory of finite-deformation viscoelasticity for elastomers and with the adoption of the Brillouin function, this work proposes a modeling framework to explore the effect of the material viscoelasticity on the dielectric permittivity of dielectric elastomers. First, the capability of the model to capture typical experimental phenomena is examined. It is found that the stretch-dependence of the dielectric permittivity can be well described by the proposed model with consideration of the material viscoelasticity. Second, the influences of the volume fraction *χ* and the relaxation time *τ* (typical parameters that reflect the viscoelasticity of the material) are also studied. Results show that the viscous network can mitigate the deformation-dependence of the dielectric permittivity while this mitigation effect weakens for a higher relaxation time. Moreover, the mechanical rate-dependence of the dielectric permittivity is modeled. The loading rate affects the dielectric permittivity mainly through the inelastic deformation (*λ*^i^) and thus *J*_v_. As the loading rate decreases, the range of the variation of the dielectric permittivity becomes narrower. Furthermore, the variation of the dielectric permittivity under cyclic loading conditions is investigated. Steady cycles of the inelastic stretch ratio, constraint *J*_v,_ and dielectric permittivity can be achieved if sufficient time is given. In addition, a different loading frequency leads to a different response pattern of the dielectric permittivity, which could be further exploited for particular applications. With the modeling framework proposed in this work, the dielectric permittivity of DEs can be more accurately predicted, especially when they are under (dynamic) mechanical constraints. Therefore, the results of this work are anticipated to provide useful guidelines for the optimal design of dielectric elastomer-related devices.

## Figures and Tables

**Table 1:** Dielectric permittivity of VHB 4910 elastomers under equi-biaxial load.

Experiment	*λ* = 1	*λ* = 3	*λ* = 4	*λ* = 5
Jean-Mistral et al. [12]	5.4	4.95	4.6	—
Wissler and Mazza [18]	4.68	3.71	3.34	—
Kofod et al. [16]	4.7	4.55	4.5	—
Tröls et al. [19]	4.12	3.64	—	3.29

## Data Availability

Data are contained within the article.
